# The Ontology for Parasite Lifecycle (OPL): towards a consistent vocabulary of lifecycle stages in parasitic organisms

**DOI:** 10.1186/2041-1480-3-5

**Published:** 2012-05-23

**Authors:** Priti P Parikh, Jie Zheng, Flora Logan-Klumpler, Christian J Stoeckert, Christos Louis, Pantelis Topalis, Anna V Protasio, Amit P Sheth, Mark Carrington, Matthew Berriman, Satya S Sahoo

**Affiliations:** 1 The Kno.e.sis Center, Department of Computer Science and Engineering, Wright State University, Dayton, OH, USA; 2 Center for Bioinformatics, Department of Genetics, University of Pennsylvania, 1420 Blockley Hall, 423 Guardian Drive, Philadelphia, Pennsylvania, 19104, USA; 3 The Wellcome Trust Sanger Institute, Wellcome Trust Genome Campus, Hinxton, Cambridge, CB10 ISA, UK; 4 Department of Biochemistry, University of Cambridge, Tennis Court Road, Cambridge, CB2 1QW, UK; 5 Institute of Molecular Biology and Biotechnology, Foundation for Research and Technology-Hellas, and Dept. of Biology, University of Crete, 700 13, Heraklion, Crete, Greece; 6 Division of Medical Informatics, School of Medicine, Case Western Reserve University, Cleveland, OH, USA

## Abstract

**Background:**

Genome sequencing of many eukaryotic pathogens and the volume of data available on public resources have created a clear requirement for a consistent vocabulary to describe the range of developmental forms of parasites. Consistent labeling of experimental data and external data, in databases and the literature, is essential for integration, cross database comparison, and knowledge discovery. The primary objective of this work was to develop a dynamic and controlled vocabulary that can be used for various parasites. The paper describes the Ontology for Parasite Lifecycle (OPL) and discusses its application in parasite research.

**Results:**

The OPL is based on the Basic Formal Ontology (BFO) and follows the rules set by the OBO Foundry consortium. The first version of the OPL models complex life cycle stage details of a range of parasites, such as *Trypanosoma* sp., *Leishmania*sp., *Plasmodium* sp., and *Shicstosoma* sp. In addition, the ontology also models necessary contextual details, such as host information, vector information, and anatomical locations. OPL is primarily designed to serve as a reference ontology for parasite life cycle stages that can be used for database annotation purposes and in the lab for data integration or information retrieval as exemplified in the application section below.

**Conclusion:**

OPL is freely available at http://purl.obolibrary.org/obo/opl.owl and has been submitted to the BioPortal site of NCBO and to the OBO Foundry. We believe that database and phenotype annotations using OPL will help run fundamental queries on databases to know more about gene functions and to find intervention targets for various parasites. The OPL is under continuous development and new parasites and/or terms are being added.

## Background

Parasitic diseases cause a burden throughout the world [1], but most importantly in the tropics and subtropics. The protozoan parasite that causes malaria remains a major threat to global health. In addition to this, important vector-borne parasites include the protozoa that cause American trypanosomiasis or Chagas disease, leishmaniasis, Human African Trypanosomiasis (HAT) or sleeping sickness, and the water-borne helminth that causes Schistosomiasis (also known as Bilharzia) [2]. Currently, treatment for many of these parasitic diseases is far from ideal. The availability of genome sequences has become central to research on the biology of these parasites and has reinvigorated the aim of identifying novel intervention targets [3-5]. The integration and mining of available data resources is a key challenge to achieve this goal and several projects, such as the T. cruzi SPSE [6], EuPathDB [7], and VectorBase [8], are focused on creating effective integration platforms for parasite and vector datasets.

The complexity of parasites and their lifecycle stages requires the use of expressive and well-defined representation formats that can be consistently and unambiguously interpreted. To illustrate the complexity of parasite lifecycle stages we consider the *Trypanosoma cruzi* parasite, which has three stages, namely amastigote, epimastigote, and trypomastigote. The amastigote is an intracellular form that is found within nucleated cells of human/vertebrate hosts of the parasite, the epimastigote is found in the midgut of an insect vector and the trypomastigote is found in the bloodstream of a vertebrate host. Further, similar lifecycle stages in different organisms may have different locations and vectors. For example, the epimastigote stage of *T. cruzi* occurs in the midgut of the triatomine kissing bug, but in *T. brucei* one type of epimastigote occurs in the salivary gland of the tsetse fly, *Glossina morsitans,* whereas another is present in the proventricles.

In addition to the complexity of modeling parasite life cycle stages, the parasitic protozoa datasets are available from multiple genome specific databases. For example,RMgmDB [9], PlasmoDB [10], andGeneDB [11], for *Plasmodium* sp; GeneDB, TriTrypDB [12], Trypsproteome [13], VSGDB [14], and TrypanoCyc [15] for the kinetoplastids, and EupathDB [16], CryptoDB [17], or GeneDB for other parasitic protozoans, such as *Cryptosporidium* sp., and *Eimeria* sp. There are now at least 5 different open access databases for *T. brucei *[11-14,18] that exist solely to capture and store experimental results. Furthermore, there are several additional databases that have information on species-specific phenotypes and many datasets produced by individual laboratories that are freely available. Each of these data sources represents parasite information, and insufficient and/or distinct annotations make it difficult to effectively integrate, query and retrieve relevant data.

Ontologies are being increasingly used to address the issue of data heterogeneity in biomedical research. Ontologies mitigate terminological heterogeneity, ensure consistent interpretation of terms through use of formal logic languages, and also facilitate automated discovery of implicit knowledge in large datasets. For example, The Gene Ontology (GO) [19] has enabled a standardized, cross-database, description of gene products. However, until recently, there has not been a consistent vocabulary for the description of lifecycle stages in parasitic organisms. In addition, availability of limited parasite lifecycle and related terms for the bioinformatics tools make it challenging in analyzing parasite datasets and pose a barrier for linking these tools together. Thus, the Infectious Disease Ontology (IDO) project (http://infectiousdiseaseontology.org/page/Main_Page) has initiated development of a family of ontologies including one for vector borne diseases that already includes an extension covering malaria [20].

The Ontology for Parasite Lifecycle (OPL) complements the efforts of the IDO project by developing an ontology that models complex details of parasite lifecycle stages including location within the host at each lifecycle stage (within tissue cells of a vertebrate host or midgut of an insect vector, etc.). OPL also describes host and vector information and is comprised of rich computational logics that would help query over multiple data repositories as shown in the Application section below. The current version of OPL covers the parasites that cause Malaria, Chagas disease, Leishmaniasis, Human African Trypanosomiasis, and Schistosomiasis. Like other ontologies, OPL is also under continuous development and it will later cover additional eukaryotic parasites within the GeneDB and EuPathDB databases, to enable consistent curation and annotation across all of the parasite genome databases currently maintained by these projects.

In this paper, we describe the current state of OPL and its applications in parasite research. We provide examples of how this ontology can be used to annotate databases and literature, and for data integration and querying. The current version of OPL (version 2.0) has 339 classes including imported as well as 233 new OPL terms that were created (as shown in Table 1). OPL reuses terms from other OBO Foundry candidate ontologies, such as the Ontology for Biomedical Investigation (OBI) [21], the Sequence Ontology (SO) [22], the Malaria Ontology (IDOMAL) [20], etc. Since OPL shares the common upper level ontology BFO and common set of relations from the Relation Ontology [23], it is interoperable with other OBO foundry ontologies. OPL is submitted to the BioPortal site of the National Center for Biomedical Ontologies (NCBO) [24] for public use and the OBO Foundry [25]. Below we provide an overview of the ontology and describe important concepts and their relationships that form the core part of OPL.

**Table 1:** Statistics of OPL specific terms and imported terms from different resources

**Ontology**	**Class Number**
Ontology for Parasites Lifecycle (OPL)	233
Basic Formal Ontology (BFO)	39
NCB ITaxonomy (NCBITAXON)	22
Uber anatomy ontology (UBERON)	14
Infectious Disease Ontology (IDO)	5
Cell Type Ontology (CL)	3
Malaria Ontology (IDOMAL)	3
Brend a Tissue Ontology (BTO)	2
Common Anatomy Reference Ontology (CARO)	2
Ontology for Biomedical Investigations (OBI)	2
Information Artifact Ontology (IAO)	6
Obo In Owl	6
owl	2
Total	339

## Results

### OPL modeling and class details

#### Modeling of lifecycle stages

**Figure 1:**
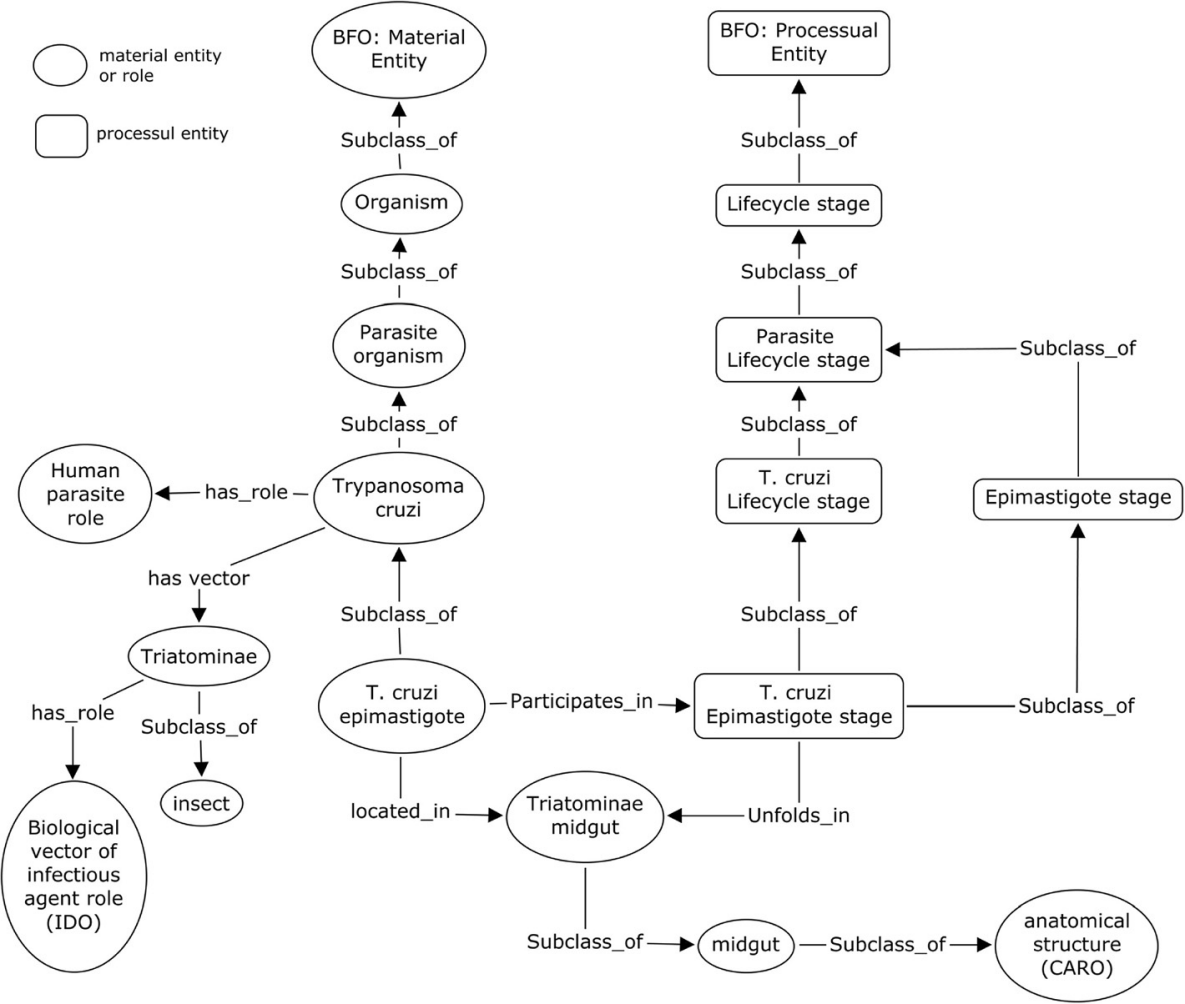
OPL Schema.

**Figure 2:**
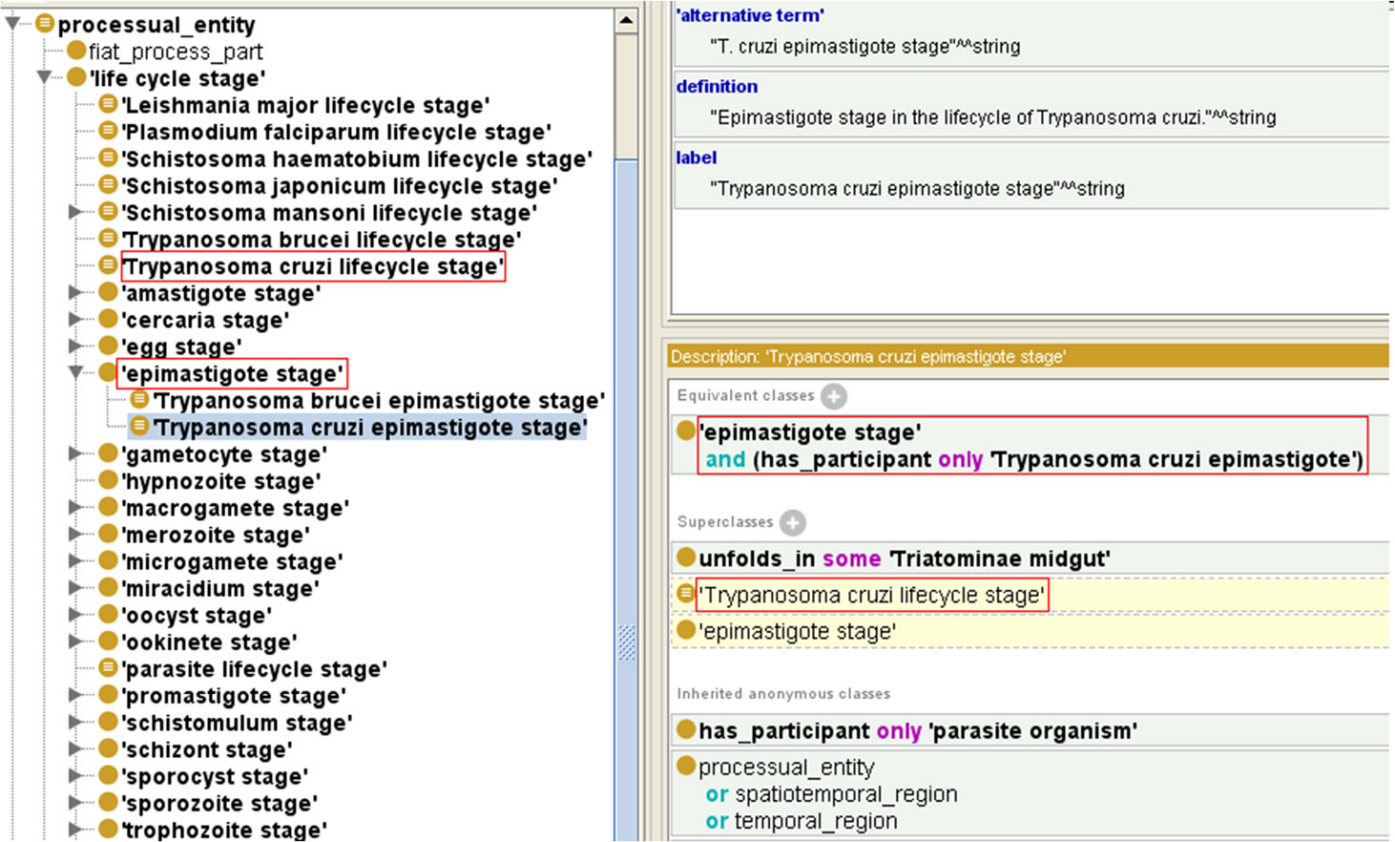
Modeling of Parasite specific lifecycle stages in OPL.

The OPL terms are represented in single quotes (‘’) throughout this report. The UBERON: ‘lifecycle stage’ concept was imported and used as the root of all lifecycle stages created in OPL. Modeling generic lifecycle of all the parasites was intricate since it involved various stages and sub-stages. Moreover, there are at least two possible ways to describe lifecycle stages; (i) generic lifecycle stages, such as sporozoite stage or epimastigote stage, and (ii) organism-specific lifecycle stages, such as *P. falciparum* sporozoite stage, *T. cruzi* epimastigote stage or *T. brucei* epimastigote stage. The organism-specific lifecycle stages provides clear information that ‘*P. falciparum* sporozoite stage’ > has_participant > only ‘*P. falciparum* sporozoite’ and not any other kind of parasitic species. This type of modeling will help avoid any wrong assumption at a later stage since the term ‘sporozoite stage’ can be applied to other parasitic species (even non-*Plasmodium*) as well. However, describing generic lifecycle stages is also important since more than one organism may have a sporozoite stage in their lifecycle. Therefore, OPL created generic lifecycle stage terms under the class UBERON: ‘lifecycle stage’. OPL also modeled parasite specific lifecycle stages under the specific class ‘Parasite lifecycle stage’. This subdivision of parasite’s lifecycle was defined as UBERON: ‘lifecycle stage’ and has_participant only ‘parasite organism’ (Figure 1). Parasite specific lifecycle stages were created in the same pattern using the has_participant relation. For example, the (‘*T. cruzi* epimastigote stage’ was defined as ‘epimastigote stage’ and has_participant *only* ‘*Trypanosoma cruzi* epimastigote’) (Figure 2). Through this logical definition, we avoid the issue of asserted multiple-inheritance. As shown in Figure 1, ‘*T. cruzi* epimastigote stage’ is an ‘epimastigote stage’ and a ‘*T. cruzi* lifecycle stage’ as well. It was asserted as an ‘epimastigote stage’ and inferred as a ‘*T. cruzi* lifecycle stage’ after reasoning (Figure 2).

Like other processes, lifecycle stages require location information as well. It is very important to model the location of the lifecycle stage since different stages may have different hosts or may occur in different anatomical structures of the same host. For example, the *T. cruzi* epimastigote stage occurs in the midgut of the *Triatominae* and the *T. brucei* epimastigote stage occurs in the salivary gland of *Glossina* flies. To capture such facts, we used the unfolds_in relationship that is used between a ‘processual_entity’ and a ‘continuant’ [23] to describe ‘*T. cruzi* epimastigote stage’ > unfolds_in > ‘*Triatominae* midgut’. For this purpose, terms for organism-specific anatomical structures were also created as mentioned in the section below.

#### Modeling of parasite organisms and anatomical structures

Two subclasses of Material Entity, Organism and Anatomical Structure are imported from OBI and Common Anatomy Reference Ontology (CARO) [26], respectively. Some subclasses of Organism are imported from the IDOMAL and NCBI taxonomy (http://purl.bioontology.org/ontology/NCBITaxon). However, new OPL-specific classes have been created under specific organisms to describe the organism in a particular lifecycle stage, such as ‘*T. cruzi* epimastigote’, ‘*P. falciparum* sporozoite’, etc. (Figure 1). Further, the location of the parasite in a particular lifecycle stage was captured using the located_in property used between two continuants [23]; i.e., ‘*T. cruzi* epimastigote’ > located_in > some ‘*Triatominae* midgut’. A connection of the parasite to the lifecycle stage, however, was captured using participates_in property. For example, ‘*T. cruzi* epimastigote’ > participates_in > only ‘*T. cruzi* epimastigotestage’.

Similarly, new classes for anatomical structures were created for specific organisms, for example, ‘*Anopheles* salivary gland’ or ‘*Homo sapiens* hepatocyte.’ This was done to avoid any confusion in the similarity of anatomical structures of various organisms. Many times insect anatomical structures are made out of only one kind of tissue in contrast to vertebrates, in which there are more than one tissue or cell types present in a typical organ. Thus, to capture the difference between the mosquito salivary gland and human salivary gland, anatomical structures were classified into organism-specific structures. Such concepts were modeled using part_of property. For example, the term ‘Anopheles salivary gland’ was defined as “UBERON: Salivary Gland and (part_of some NCBI Taxon: Anopheles).” The class ‘Salivary Gland’ was imported from UBERON [27] since it defines salivary gland in general and not any species specific anatomical structure. Further, OPL captures restrictions between parasites and their hosts using located_in property. For instance, ‘*P. falciparum*sporozoites’ > located in > some (‘Anopheles salivary gland’ or ‘human hepatocyte’). Finally, some classes under Anatomical Structure are imported from the Cell type Ontology (CL) [28] and BRENDA Tissue Ontology (BTO) [29].

### Applications/use cases

#### Database annotations and queries

**Figure 3:**
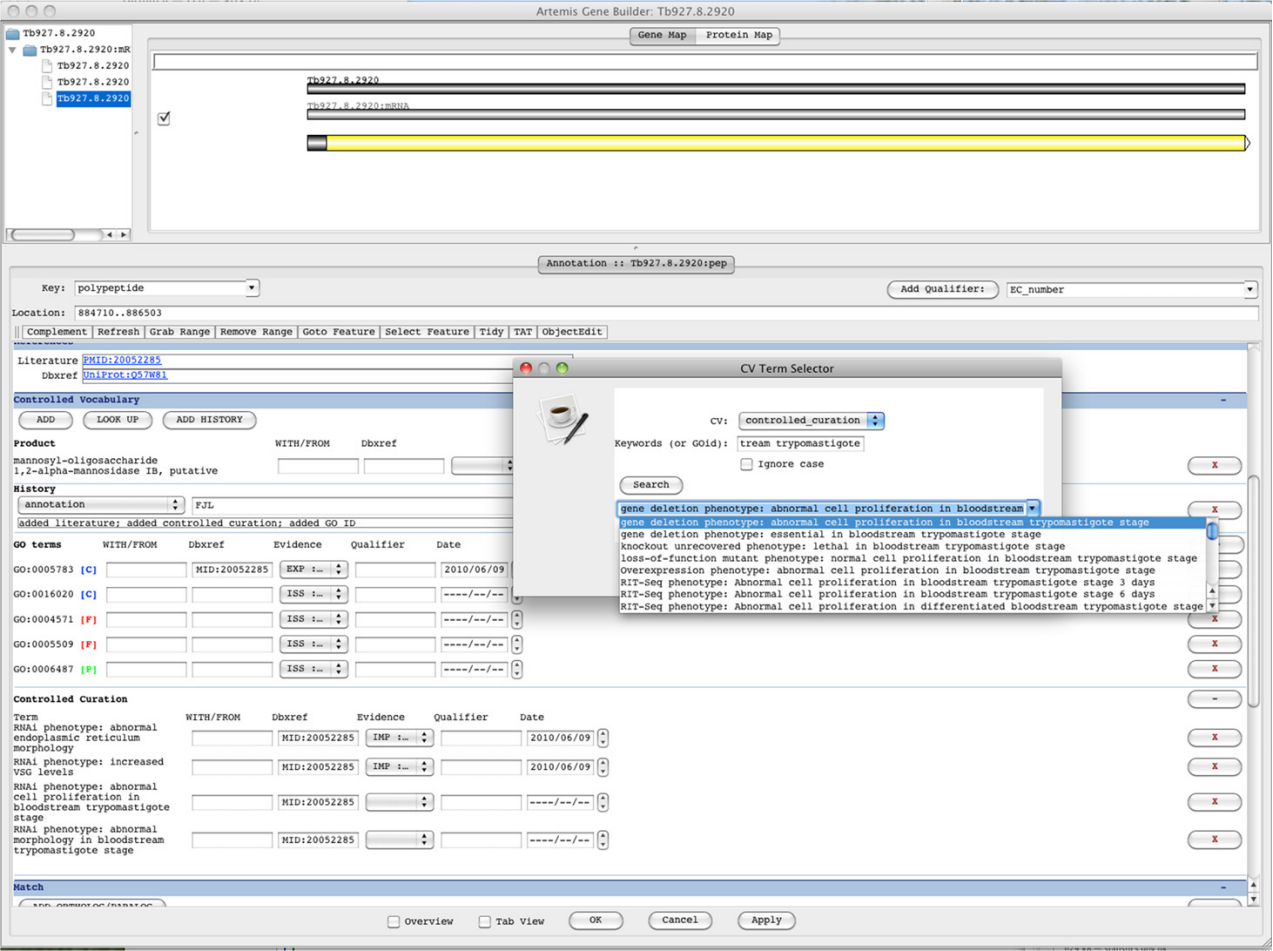
Use of OPL for Annotations of Parasite Lifecycle Stages in GeneDB.

The Pathogen Genome database (GeneDB) [11] is responsible for the active curation and annotation of several parasitic organism reference genomes, namely *Trypanosoma brucei*, *Trypanosoma cruzi*, *Leishmania major*, *Plasmodium falciparum*, and *Schistosoma mansoni*, as well as curating and annotating the genomes of numerous related organisms. The annotated genomes are then used by databases such as EuPathDB to be integrated with a wide variety of functional genomics datasets made available by members of the global research community, and thus accurate and consistent annotation is essential. The OPL is being used by the Wellcome Trust Sanger Institute (WTSI) to annotate their GeneDB that will help run structured queries on the database (Figure 3). One example query is, “list all the genes that encode a surface protein in *P. falciparum* sporozoite stage.” Running such queries on the database will help researchers to answer fundamental questions on gene function. Currently, all annotations that include a lifecycle stage description fall into the category of phenotype curation.

Phenotype annotations are a key aspect of the curation process, in which experimental results are entered into the database in a standardized format. The new controlled curation system draws terms from several ontologies to create a statement about a phenotype that is associated with a gene model. Since the majority of experiments carried out in parasitic organisms are done in a single lifecycle stage, it is important to annotate phenotype data with this information to enable users to analyze the data appropriately. Further, there is a wide range of terms used to describe lifecycle stages within each parasite species, which makes searching the phenotype curation more complex than it needs to be. The OPL, however, now provides standardized terms for lifecycle stages across all of the genomes that are actively curated by GeneDB. OPL will be combined with other available ontologies, such as GO, to add a depth of information to curations, enabling users to identify not only the lifecycle stage in which a phenotype was observed, but also the cellular compartment and the biological process with which it is associated. Examples of GeneDB phenotype annotation in *T. brucei*, which can utilize OPL include:

A. *T. brucei* bloodstream form trypomastigote stage

1. Tb927.10.10390 Trypanothione Reductase.

Current curation = loss-of-function mutant phenotype: lethal during bloodstream stage.

2. Tb927.8.2210 Pteridine Reductase.

Current curation = RNAi phenotype: essential in bloodstream form.

3. Tb927.7.5930 Protein Associated With Differentiation.

Current curation = expressed in stumpy form.

B. *T. brucei* procyclic trypomastigote stage

4 Tb927.5.2900 Histone Deacetylase 4.

Current curation = conditional null mutant phenotype: essential for fly midgutcolonisation.

5 Tb11.02.2260 MCAK-like kinesin, putative.

Current curation = gene deletion: no effect on growth in procyclic form

6. Tb927.3.4290 Paraflagellar Rod Protein.

Current curation = RNAi: decreased cell motility during procyclic stage.

In both of these examples, the OPL term will be used to replace the ambiguous and inconsistent terms currently in use for annotation. As a team of curators carries out phenotype annotation manually, OPL usage will remove any ambiguity and inconsistency in the descriptions of lifecycle stages associated with phenotypes. EuPathDB will also use OPL for annotation of investigations in which lifecycle stages represent an important context [30]. To address community needs, EuPathDB collects datasets for a wide variety of eukaryotic parasite species from both individual investigations and external resources. OPL and other ontologies that follow OBO Foundry principles (such as GO and OBI) will be used to provide consistent representation of experimental contexts such as the lifecycle stage for which phenotype data was collected. Consistent usage of terms from OPL and other ontologies will facilitate sharing and exchanging data among different resources, integration of data from external resources, such as genome data from GeneDB, and data exploration and analysis.

#### Literature annotation

**Figure 4:**
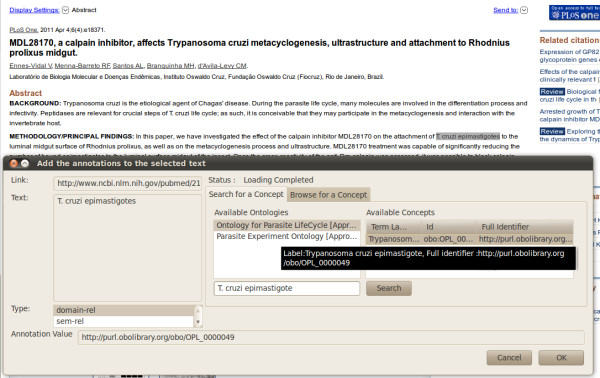
Literature Annotation of Lifecycle Terms using OPL.

Other than genomics databases, such as EuPathDB or GeneDB, a large volume of experimental biological information is also buried in the fast-growing literature databases, such as PubMed. Therefore, research institutes like the WTSI have initiated efforts to annotate scientific literature using ontologies. They currently use Artemis [31], which provides a platform to annotate the scientific literature using GO. Now they will be able to annotate parasite lifecycle stages and other details using OPL. To annotate the lifecycle stages and other details in the literature either Artemis can be extended to include OPL, or another tool, such as Kino [32], can be used for this purpose. Kino is a document management system that researchers at the Ohio Center of Excellence for Knowledge-enabled Computing (Kno.e.sis) at the Wright State University developed along with WTSI using SA-REST and faceted search. Kino allows biologists to annotate any web documents using ontologies listed at the NCBO BioPortal, submit them to a Kino repository, and search them using ontology terms or synonyms (Figure 4). Currently, WTSI is evaluating their existing system and Kino for the use in annotating PubMed literature in parasite domains that will utilize OPL.

#### Data integration and knowledge discovery

OPL is currently being used in the Semantic Problem Solving Environment (SPSE) for *T. cruzi* project along with the Parasite Experiment Ontology (PEO) [33,34]. *T. cruzi* SPSE is a collaborative effort among researchers at the Kno.e.sis Center at the Wright State University, the Center for Tropical and Emerging Global Diseases (CTEGD) at the University of Georgia, and the National Center for Biomedical Ontologies (NCBO) at the Stanford University. The primary objective of this research is to develop a comprehensive and intuitive environment using semantic web technologies that will facilitate the identification of vaccine, diagnostic, and/or gene knockout targets in *T. cruzi* and related parasites. For this purpose, OPL, along with PEO, were used as reference ontologies to convert the data into a Resource Description Framework (RDF) format and semantically integrate data from various information sources, such as pathway, ortholog, and gene information resource. PEO was also used to develop forms to capture the provenance data in the biology lab. These forms use a user-friendly interface and generate the output data in RDF format based on ontology schema. OPL can be used effectively for this purpose and also to provide consistent labeling of parasite research data that involves lifecycle stages. The forms that use ontologies as schema facilitate consistent labeling of the data and make data integration easier at later stages. The client can either use SPARQL queries or a query processing tools like Cuebee [35] to query such semantically integrated datasets and find new knowledge such as intervention targets for parasites. Some example queries that utilize OPL for this purpose are:

1) Compare the gene expression profile of different parasites that are found in human macrophages.

2) Compare all the *P. falciparum* genes that are expressed during vector-based lifecycle stages and not during vertebrate-host lifecycle stages.

3) Show all the enzymes expressed during the slender trypomastigote stage in the glycosome and not expressed in the procyclic stage.

4) List all the *T. cruzi* genes that are expressed in amastigote stage only. Also, how many of these genes are involved in only one metabolic pathway.

Some databases including EuPathDB do not collect information regarding parasite vectors and anatomical locations in a structured manner. OPL will be very important for such databases and it will enable users to query the databases for such information (please see Query 1 and 2), by providing an additional, potential interoperability tool between, for example, EuPathDB and VectorBase. OPL can also be used along with other ontologies, such as GO or PEO to find vaccination, drug, or gene knockout targets in parasites (Query 3 and 4) [34].

## Discussion and conclusion

OPL is a collaborative effort among national and international institutes with expertise in parasite and vector research, ontology development, semantic web, and bioinformatics. OPL describes complex lifecycle stage details of various parasites along with their host, vector, and anatomical location information. Since the sequences of many parasite genomes are available publicly on GeneDB, GenBank, and EuPathDB, annotation of gene products has become a priority. Moreover, consistent labeling of parasite data including external databases, experimental data, and literature, is essential in data integration, cross database comparison, and uniform data access. Currently the only aspect of annotation that is structured uses the Gene Ontology (GO) in conjunction with an evidence code that describes the experiment type used to generate the data. This gives users the ability to search the database according to GO annotations. Since not every phenotype can be described using GO terms alone, GeneDB is building a controlled phenotype curation system that will utilize GO in combination with other relevant ontologies. For such a curation system, the annotation of parasite lifecycle stages for the experimental data is very desirable to GeneDB and EuPathDB users. Since a lifecycle ontology for parasitic organisms was not already available, the creation of OPL is expected to have a significant impact on the curation process of parasite data. Using OPL and other ontologies, the vast wealth of parasite knowledge can be used effectively by parasite experts, database developers, and technicians developing decision support tools to control such diseases. Moreover, such ontologies can be used to structure the parasite phenotype data and to allow queries on these databases. The data integration and annotations using OPL also helps parasite researchers to (i) advance their understanding of gene function, (ii) interpret functional genomics datasets, (iii) query phenotypes to find intervention targets, and (iv) facilitate integration of various data sources as exemplified in the Application section above.

OPL complies with the OBO Foundry principles and reuses many terms from existing OBO Foundry ontologies, such as OBI, CL, CARO, UBERON, and BTO. As more data annotated using OPL become available, its utility to the broader parasite research community will continue to increase. The latest version of OPL (current is v 2.0) is always available at http://purl.obolibrary.org/obo/opl.owl and also posted on BioPortal site of NCBO (http://purl.bioontology.org/ontology/OPL) for public use.

In summary, OPL represents a significant effort to model the complex lifecycle of various parasites including information on their host, vector, and anatomical location. As shown in the application and use cases, we believe that consistent labeling of parasite data and database annotations using OPL will facilitate data integration and uniform data access, and also help parasitologists answer the fundamental question of *what a gene product does in a specific lifecycle stage*.

## Method

OPL is developed using the Basic Formal Ontology (BFO) [36] as upper level ontology and follows the principles set by the OBO Foundry consortium [37]. OPL is expressed in the W3C standard Web Ontology Language (OWL) 1.0 as it supports richer semantics than the Open Biomedical Ontologies (OBO) format, another commonly used language in the biomedical domain. The OWL Description Logic (OWL-DL) was chosen to gain maximum expression and support for the automated reasoning, inferences, and consistency-checking are important for ontology development and maintenance. The meta-data schema of OPL is implemented as OWL annotation properties defined in the Information Artifact Ontology (IAO, http://purl.obolibrary.org/obo/iao), which have been widely used by many OBO foundry ontologies, such as IDO [38] and OBI. Two key external ontologies, BFO and IAO meta-data part, are directly imported into OPL using the owl:import mechanism which imports the whole ontologies.

To eliminate the redundant efforts and ensure orthogonality, OPL maximizes the use of existing ontologies already listed by the OBO foundry. Since importing the whole ontology is impractical, Minimal Information Reference External Ontology Term (MIREOT) strategy [39] is adopted to import the terms from the external resources. This strategy provides the consistency, flexibility, and scalability of referring the external resources. The minimal information of an external term includes source ontology URI, source term URI, and target direct superclass URI. The OPL mainly references terms in four reference ontologies, IDO for existing parasite related terms, NCBI taxon (http://purl.bioontology.org/ontology/NCBITaxon) for organism, Cell Ontology for cell type, and UBERON Ontology for cross species anatomical structure. OPL provides the textual definitions for all the terms and logical definitions for each term when possible. The ontology was initially developed using Protégé 4.1 (http://protege.stanford.edu/), later switching to the WebProtege ontology editor [40] since it provides a better collaborative working environment and allows the developers to track changes made by others. The reasoner tools, such as Hermit (http://hermit-reasoner.com/) or Pellet [41], were used for consistency checking and inferences.

The terms in OPL were collected from our collaborators and the community members who intend to use OPL. The ontology will continue to expand to meet the needs of a parasite research community to cover more eukaryotic parasites and their lifecycle stages. The BioPortal or Trykipedia site (http://wiki.knoesis.org/index.php/Trykipedia) will be used to collect more terms in the future. The current developmental version of OPL is available at: http://webprotege.stanford.edu/#OPL. We plan to release new inferred versions of OPL quarterly, if required, to provide any new updates on the ontology. During the release process, the permanent OPL identifiers will be assigned and the compliance with OBO policies will be checked. The releases of OPL will also be uploaded to both the NCBO BioPortal and the OBO Foundry repository for public use.

## Availability of supporting data

The web Protégé link for OPL development, http://webprotege.stanford.edu/, shows the change history and developers’ comments, discussion, and the reason for changes made.

The queries and results of the Semantic Problem Solving Environment (SPSE) for *T. cruzi* project where OPL was used along with other ontologies can be found here: http://wiki.knoesis.org/index.php/Manuscript_Details.

## Competing interests

The authors declare that they have no competing interests.

## Authors’ contributions

PPP contributed to the development of the ontology, created/edited textual definitions of ontology terms, and wrote and edited the manuscript. JZ and FLK contributed equally in this work. JZ helped develop ontology making sure its compliance with OBO foundry principles, maintained the ontology on WebProtege for better collaboration, took responsibility for OPL release, and helped write the manuscript. FLK provided ontology terms and textual definitions, and helped develop the ontology and write the manuscript. CJS, CL, PT contributed to the formal ontological structure of OPL, improved textual definitions, and edited the manuscript. AP obtained funds, provided scientific direction, and critically reviewed and edited the manuscript. MC and MB provided textual definitions and reviewed the manuscript. SSS was involved in the ontology development, and critically reviewed and edited the manuscript. All authors read and approved the final manuscript.
